# The Adenosinergic System in Diabetic Retinopathy

**DOI:** 10.1155/2016/4270301

**Published:** 2016-02-29

**Authors:** J. Vindeirinho, A. R. Santiago, C. Cavadas, A. F. Ambrósio, P. F. Santos

**Affiliations:** ^1^Center for Neuroscience and Cell Biology (CNC), University of Coimbra, 3004-504 Coimbra, Portugal; ^2^Institute for Interdisciplinary Research (III), University of Coimbra, 3030-789 Coimbra, Portugal; ^3^CNC.IBILI, University of Coimbra, 3004-504 Coimbra, Portugal; ^4^Institute for Biomedical Imaging and Life Sciences (IBILI), Faculty of Medicine, University of Coimbra, 3000-548 Coimbra, Portugal; ^5^Association for Innovation and Biomedical Research on Light and Image (AIBILI), 3000-548 Coimbra, Portugal; ^6^Faculty of Pharmacy, University of Coimbra, 3000-548 Coimbra, Portugal; ^7^Department of Life Sciences, Faculty of Sciences and Technology, University of Coimbra, 3000-456 Coimbra, Portugal

## Abstract

The neurodegenerative and inflammatory environment that is prevalent in the diabetic eye is a key player in the development and progression of diabetic retinopathy. The adenosinergic system is widely regarded as a significant modulator of neurotransmission and the inflammatory response, through the actions of the four types of adenosine receptors (A_1_R, A_2A_R, A_2B_R, and A_3_R), and thus could be revealed as a potential player in the events unfolding in the early stages of diabetic retinopathy. Herein, we review the studies that explore the impact of diabetic conditions on the retinal adenosinergic system, as well as the role of the said system in ameliorating or exacerbating those conditions. The experimental results described suggest that this system is heavily affected by diabetic conditions and that the modulation of its components could reveal potential therapeutic targets for the treatment of diabetic retinopathy, particularly in the early stages of the disease.

## 1. Introduction

Diabetic retinopathy (DR) is the most common complication of diabetes [1] and one of the leading causes of visual impairment and preventable blindness in working age adults in the world [2, 3]. In fact, DR is highly prevalent in both type 1 and type 2 diabetic patients: nearly 77% of type 1 diabetic patients present some degree of the disease [3], and over 60% of type 2 diabetic patients (T2DM) after 20 years with diabetes present retinopathy to some degree [4]. The predominant view of DR has been that diabetes primarily affected the retinal microvasculature and this then caused secondary damage and degeneration. However, over the years, this notion has been contested, with several studies demonstrating that the neural retina is also affected early in diabetes [5], and vision impairments are detected earlier than the vascular changes [6], suggesting that neural changes are a result of diabetes and not a consequence of BRB breakdown [5]. In fact, DR shares many similarities with neurodegenerative diseases, such alterations in glutamatergic system [7–11], apoptosis [12–15], glial activation [6], and inflammation [16–19]. Studies showed a correlation between the toxic levels of extracellular glutamate and the increased damage and higher apoptotic levels in retinal neurons observed in diabetic conditions [20, 21]. The altered levels of several neurotrophic factors in diabetic conditions can also exacerbate the damage occurring in retinal neurons, affect glucose metabolism, and contribute to an inflammatory environment [22–25]. This inflammatory environment has characteristics that are usually associated with chronic inflammatory conditions such as an increase in leukostasis, microglial cell activation, activation of NF-*κ*B leading to the amplified production and release of proinflammatory cytokines, chemokines, and other inflammatory mediator proteins [6, 26, 27]. Cytokines such as TNF, interleukin-1*β* (IL-1*β*), and interleukin-6 (IL-6) are increased in DR and connected to retinal leukostasis, BRB breakdown, and the higher levels of apoptosis present in DR [16, 17, 19, 28].

## 2. The Retina

The retina is a part of the central nervous system (CNS) that lines the inner, posterior surface of the eye, containing four types of cells: neuronal cells, macroglial and microglial cells, and blood vessel cells. There are several types of neuronal cells in the retina: photoreceptors, bipolar, amacrine, horizontal, and ganglion cells. Photoreceptors mediate phototransduction, while a complex signaling network existing between the different neurons modulates and communicates nerve impulses. These are then transmitted to the brain across the axons of retinal ganglion cells that form the nerve fiber layer and optic nerve. The two main types of macroglial cells present in the retina are Müller cells and astrocytes, and they are involved not only in retinal metabolism but also in the modulation of the activity of both neurons and blood vessels. Müller cells cross the width of the neural retina, forming a connection between retinal neurons, the vitreous body, and blood vessels, by being key regulators of neuronal function, extracellular ionic balance, and glutamate metabolism. Astrocytes are present in the nerve fiber layer and their processes wrap blood vessels and retinal ganglion cells. Macroglial cells are then vital in the communication between the vascular and the neuronal parts of the retina. Microglial cells are the resident immune cells of the central nervous system, surveying the retinal parenchyma and being highly sensitive to alterations in the homeostatic environment. The blood vessels are composed of pericytes, involved in the regulation of vascular flow, and endothelial cells, vital for hemostatic functions and the formation of the blood-retinal barrier (BRB). The activity of these cells and the interaction between them are necessary for normal retinal function, and if their normal functions are impaired, this can result in loss of visual capacity [29, 30].

## 3. The Adenosinergic System

Adenosine is a purine nucleoside essential to all living cells. Beyond its role in metabolism and genetic transmission of information, adenosine is also a messenger that regulates numerous physiological processes in several tissues, particularly in the cardiovascular and nervous systems. Studies have shown the importance of adenosine in both normal and pathological situations, from regulation of sleep and linking energy demands to cerebral blood flow to inflammatory responses and in neuroprotection against ischemia and epilepsy [31–33]. In the retina, most knowledge of adenosine function is based on research in pathological contexts, but nevertheless, a picture is forming of a signaling system involved in neurotransmission modulation [34], vascular processes [35], control of osmotic alterations [36], and inflammatory response [37], as a background modulatory system, or in transient responses to alterations to the homeostasis.

Several components are involved in this regulatory system: the actions of adenosine are mediated via four G protein-coupled receptors (GPCR), namely, the A_1_, A_2A_, A_2B_, and A_3_ adenosine receptors, although some G-protein-independent effects have also been reported [38, 39]. The adenosine receptors are abundant throughout the organism, but particularly in the CNS where the receptors can be found in all brain areas [32]. In the retina, most neuronal cells express A_1_R and/or A_2A_R, with retinal ganglion cells also expressing A_3_R [40–42]; Müller cells have all but one receptor type present, with no direct A_3_R detection so far [43], while astrocytes and microglial cells express all four receptors [44–47]; pericytes express A_1_R and A_2A_R and endothelial cells express A_2A_R and A_2B_R [48, 49] while epithelial cells can express all four receptors [40, 50, 51].

A_1_R and A_2A_R have generally opposing signaling effects: A_1_R activation has a largely inhibitory action, especially in neurons, where A_1_R signaling causes a decrease in the release of neurotransmitters and overall excitability [32, 39]. A_2A_R activation, on the other hand, has a more excitatory role in neurons, being responsible for the potentiation of synaptic transmitter release [32, 39]. In other systems, however, the activation of A_2A_R can also have inhibitory actions [38].

A_2B_R and A_3_R, due to their more recent discovery and characterization, have roles that are less understood. A_2B_R, much like A_2A_R, has been linked to increased neurotransmission in the central nervous system and anti-inflammatory pathways, although some reports show a potential proinflammatory action in some circumstances [38, 39, 52].

A_3_R is probably the receptor that is less known, with several studies showing a double nature in different pathophysiological conditions, often going from protective to destructive depending on the agonist concentration or experiment time-frame [53].

The different adenosine receptors have distinct but frequently overlapping distributions. The proximity of different adenosine receptors means that adenosine can activate multiple signaling pathways and create a self-modulating environment in several cases, with A_2A_R and A_2B_R activating adenylate cyclase and A_1_R and A_3_R inhibiting it. In the case of A_2A_R and A_2B_R, the interactions may be synergistic, with the lower affinity A_2B_R supporting the higher affinity A_2A_R, which may suffer desensitization faster [39].

### 3.1. Management of Adenosine Levels

**Figure 1:**
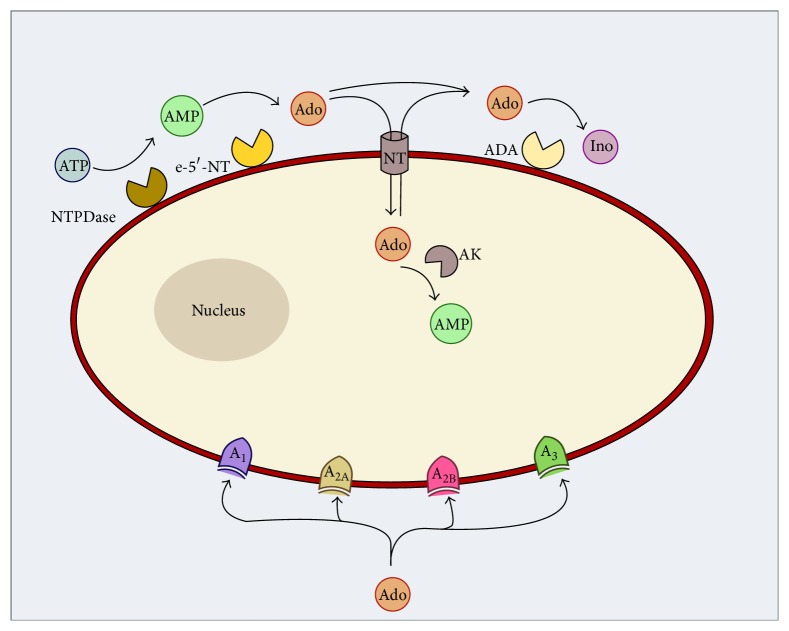
Representation of the adenosinergic system, from adenosine formation and release to signaling and removal from the extracellular space. ADA: adenosine deaminase; Ado: adenosine; AK: adenosine kinase; AMP: adenosine monophosphate; ATP: adenosine triphosphate; e-5′-NT: ecto-5′-nucleotidase; Ino: inosine; NT: nucleoside transporter; NTPDase: nucleoside triphosphate diphosphohydrolase.

The intra- and extracellular levels of adenosine are controlled by several enzymes and transporters, allowing a regulation that is fine-tuned to disturbances in homeostasis, as illustrated in Figure 1.

The release of adenine nucleotides and its posterior extracellular conversion into adenosine is one of the mechanisms that can lead to an increase in the extracellular levels of adenosine. ATP is an important source of extracellular adenosine, being released, not only in normal conditions, but also in response to different forms of stress and during necrosis or apoptosis [54]. Several extracellular enzymes handle the conversion of released adenine nucleotides: nucleoside triphosphate diphosphohydrolases (NTPDase), ecto-nucleotide pyrophosphatase/phosphodiesterases (ecto-NPP), and apyrases create a chain where adenine nucleotides can be dephosphorylated to 5′-AMP fairly quickly, which is then dephosphorylated to adenosine by 5′-ectonucleotidase (5′-eN) [31]. These enzymes are present in the CNS [31, 55], including the retina [56, 57], where Müller cells, in particular, have this enzyme chain best characterized [36, 56].

Another mechanism by which extracellular adenosine levels increase is the release of intracellular adenosine by bidirectional nucleoside transporters: equilibrative nucleoside transporters (ENTs) and concentrative nucleoside transporters (CNTs). The ENTs consist of four characterized isoforms (ENT1–4) and mediate a facilitated bidirectional diffusion of nucleosides through the plasma membrane, transporting naturally occurring nucleosides with broad selectivity. All of them are widely distributed, but ENT2 levels are higher in brain and skeletal muscle, while ENT4 has a more marked presence in heart, liver, and brain [58]. The CNTs, consisting of three isoforms (CNT1–3), perform a secondary active transport function, where the inward transport of a nucleoside is coupled with a transmembrane sodium gradient. Of the three, only CNT2 is expressed in brain [58]. As for the retina, studies have shown the expression of ENT1-2 and CNT1-2 in whole retina extracts from rats [59] and also a rat Müller cell line [60]. In a rat retinal capillary endothelial cell line, the expression of ENT1-2 and CNT2-3 was also detected [59]. Due to the presence of ENTs, the transport of adenosine can be made following concentration gradients [61], which means that the concentration of intracellular adenosine can directly affect the extracellular levels of this nucleoside, making the regulation of the intracellular enzymes involved in the adenosine cycle also an important step in the control of extracellular adenosine levels.

Beyond nucleotide degradation and transporter release, one group found evidence to support the existence of a mechanism for the direct release of adenosine in the cerebellum of mice, through adenosine-filled vesicles [62].

The nucleoside transporters are also involved in the reuptake of adenosine by neurons and neighboring cells to prevent unwanted receptor activation. Intracellularly, adenosine can be either phosphorylated by adenosine kinase (AK) to form AMP or degradated by adenosine deaminase (ADA). Both enzymes are present in rat retina [63]. Due to its lower *K*_*m*_ (2 *μ*M for AK while ADA features a *K*_*m*_ of 17–45 *μ*M, in rat whole brain) it is probable that phosphorylation by AK is the principal pathway of adenosine removal in physiological conditions, while deamination may be more relevant in pathological conditions (when adenosine concentrations rise) [31, 55].

Apart from reuptake, extracellular adenosine can also be removed by deamination to inosine by extracellular ADA, although most of the extracellular adenosine is cleared by reuptake in normal conditions [31]. During hypoxia and ischemia ecto-ADA may gain a more prominent role in the control of extracellular adenosine concentrations, since these situations raise the intracellular levels of adenosine, affecting the gradient concentration and disrupting the inward flow of nucleotides through the transporters [31].

## 4. Adenosine and Diabetic Retinopathy

Various forms of stress and pathological conditions, from ischemia and hypoxia to epilepsy can affect the adenosinergic system, hinting at a pattern of alterations to the density and distribution of adenosine receptors that is induced by such conditions [64]. Diabetes is no exception, with several studies investigating the effect of diabetes and hyperglycemia on adenosinergic system components in various tissues and models [65–68]. However, only in recent years did some studies reveal the extent of the effect that diabetic conditions have on the adenosinergic system in the retina.

Changes in the retina have been reported in retinal neural cells exposed to elevated glucose concentration and in diabetic rats. Recently, studies revealed that the expression and density of A_1_R suffer alterations in diabetic conditions in the retina: both mRNA and protein levels of this receptor are increased in rat retinal cell cultures subjected to high glucose conditions, and the density of A_1_R is also increased in rat retinas after 4 weeks of diabetes [63]; in that period the retinal mRNA levels of A_1_R are not significantly different from the control animals. However, when diabetes was sustained for a longer period of time (12 weeks) a decrease in the expression of this receptor was observed [69].

A_2A_R has become a focus of study in the diabetic retina, mainly due to its potential role in the inflammatory conditions in early diabetic retinopathy. In mouse, rat, and pig, this receptor is shown to be upregulated in diabetic conditions, both in retinal cell cultures and diabetic retinas [63, 70, 71].

As for the A_3_R receptor, one study showed that the expression and density of this receptor show a transient increase in diabetic rat retinas, after one week of diabetes, and a significant decrease after 4 weeks of diabetes [63].

The enzymes involved in the regulation of adenosine levels are also very sensitive to metabolic and homeostatic alterations. Both nucleotide degrading (NTPDases, 5′-eN) and adenosine degrading (ADA, AK) enzymes were shown to be affected by diabetes in several tissues [72–76].

A study performed in the retina of diabetic rats showed that adenosine is heavily involved in the control of osmotic glial swelling during diabetes and that diabetic conditions prompt a differential expression of the nucleotide degrading enzyme NTPDase1, which hydrolyzes both ATP and ADP in equal measure, from being restricted to blood vessels in control animals to being present in retinal glial cells in diabetic animals [36]. The same study revealed an increase in mRNA levels of NTPDase1 in diabetic retinas, while NTPDase2 and 5′-eN expression were the same.

Another factor that may affect extracellular adenosine levels is the increase in ATP release. In fact, it was reported in rat retinal neural cells cultured with elevated glucose concentration that the depolarization-evoked release of ATP increases. The same study demonstrated that ATP degradation is impaired in the same conditions [18].

In diabetic rat Müller cells, glutamate signaling can cause an increase in the extracellular generation of adenosine, through 5′-eN [36], probably due to the alterations in expression and distribution of NTPDase1, which drives an increase in AMP availability for degradation by 5′-eNT.

In diabetic rat retinas after 4 weeks ADA expression and density are decreased, while in retinal cultures exposed to high glucose the activity of this enzyme is severely decreased, which could be responsible for the high levels of extracellular adenosine found in those conditions [63]. On the other hand, a study in human and pig retinas indicates that, while the isoenzyme ADA1 remains unchanged in diabetic conditions, the protein levels of the isoenzyme ADA2 are increased. Furthermore, in porcine retinas, the activity of ADA2 is increased [71].

Although AK expression and density levels were unaltered in rat retinal cultures exposed to high glucose and in rat diabetic retinas at 1 week of diabetes, there was a decrease observed at 4 weeks of diabetes [63]. Similarly, in the diabetic retinas of mice, AK protein levels were decreased after 8 weeks of diabetes, and this decrease is reduced by the inhibition of AK itself [70].

In a study performed in T2DM patients with DR, an increase in plasma levels of adenosine was observed, but not in patients without DR, leading the authors to suggest adenosine as a biomarker for the diagnosis of DR [77].

**Figure 2:**
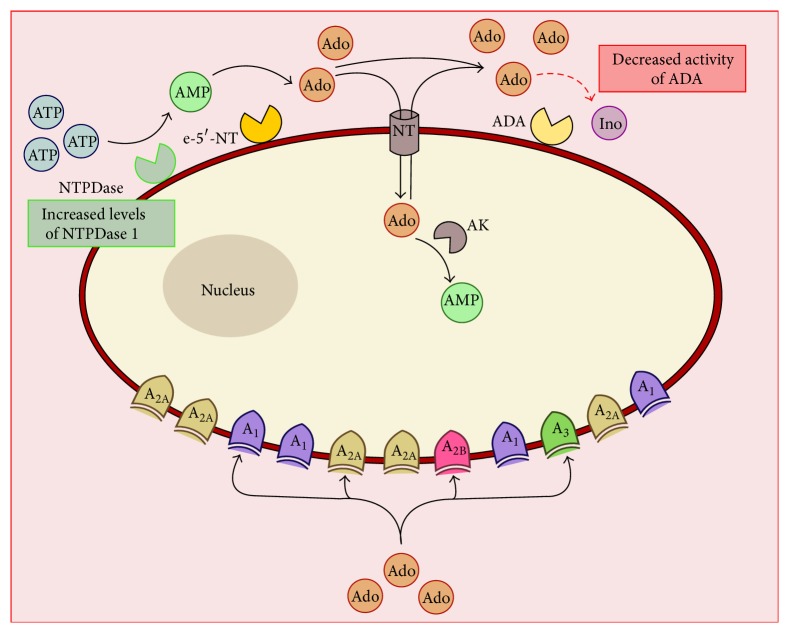
General representation of the alterations occurring to the adenosinergic system in diabetic conditions. ADA: adenosine deaminase; Ado: adenosine; AK: adenosine kinase; AMP: adenosine monophosphate; ATP: adenosine triphosphate e-5′-NT: ecto-5′-nucleotidase; Ino: inosine; NT: nucleoside transporter; NTPDase: nucleoside triphosphate diphosphohydrolase.

These alterations, as shown in Figure 2, occurring at several levels of the adenosinergic system in the diabetic retina may result in functional alterations to the processes controlled or modulated by adenosine.

The osmotic swelling of glial cells present in diabetic retinas is prevented by administration of adenosine (10 *μ*M) in rats, but this prevention is suppressed by using DPCPX, an A_1_R antagonist [36], while the blockade of A_2A_R had no effect. This suggests that the action of adenosine is mediated by activation of A_1_R, which opens potassium and chloride channels in the outer membrane of glial cells. The same study showed that the purinergic signaling that prevents swelling under osmotic stress is inactive in diabetic conditions, although the receptors involved are functional and the mechanism works upon adenosine administration (as mentioned above).

The potential anti-inflammatory action of adenosine was evaluated in the retina: in retinal microglia cultures, exposure to Amadori-glycated albumin (AGA) triggers the inflammatory response seen in diabetic conditions, namely, the increased expression and release of TNF, and this effect of AGA was successfully blocked by activation of A_2A_R in mice [78], rat [70], and pig [71] retinal microglial cells. In mice, the activation of A_2A_R with CGS21680, a selective agonist, also reduced TNF levels and decreased cell death in diabetic retinas [78].

In a study performed in diabetic mice, treatment with ABT-702, an AK inhibitor decreased ENT1 protein levels, cell death, and the expression of Iba1 (a microglia/macrophage marker that is upregulated upon activation), suggesting a decrease in microglia reactivity [70]. The same study showed that, in rat retinal microglial cell cultures, the inhibition of AK had a more substantial effect than ADA inhibition in decreasing TNF release.

These results point out to a protective, anti-inflammatory effect of adenosine and A_2A_R activation in particular. This protective effect, however, is contentious, with at least one other study showing conflicting results: the treatment of rMC-1, a rat Müller cell line, incubated in hyperglycemic-like conditions with either ATP or adenosine scavengers reduced the proinflammatory caspase-1 activation [79]. A nonselective adenosine receptor antagonist (DPCPX at a concentration of 10 *μ*M) also reduced caspase-1 activation, which was mimicked with an A_2B_R selective antagonist (MRS1754). Taken together, the existing data on the role of adenosine in the retina during diabetes remains controversial and the effects of the modulation of this system most likely will depend on the target receptors and/or cell types and be influenced by the experimental approach. However, a picture of dysregulation is already apparent, one that may impact on excitotoxicity, osmotic regulation, and inflammation, all of which can impair cell survival and visual function in diabetic conditions.

## 5. Conclusions

In this review we covered the numerous alterations that occur to the adenosinergic system in the diabetic retina, from receptor expression and enzyme distribution and activity to adenosine levels. Such modifications to a system that is highly sensible to disruptions of homeostasis will necessarily have repercussions, especially in inflammatory and excitotoxic situations. The potential protection or damage that these alterations may cause is contentious, but the modulation of this system seems to be a promising path to combating the inflammatory and excitotoxic environment and reducing cell death in early DR.
